# Differential Modulation of Cancer Cell Proliferation by Fermented Plant-Based Beverages: A Comparative Study of Tiger Nut, Carob and Rice Beverages in Colorectal Adenocarcinoma Cells

**DOI:** 10.3390/foods14173072

**Published:** 2025-08-30

**Authors:** Matteo Vitali, Mussa Makran, Mónica Gandía, Antonio Cilla, Amparo Gamero

**Affiliations:** 1 Bionutest Research Group, Food Technology Area, Faculty of Pharmacy and Food Sciences, University of Valencia, Av. Vicente Andrés Estellés s/n, 46100 Burjassot, Valencia, Spain; matteo.vitali@uv.es (M.V.); mussa.makran@uv.es (M.M.); monica.gandia@uv.es (M.G.); amparo.gamero@uv.es (A.G.); 2 Bionutest Research Group, Nutrition and Food Science Area, Faculty of Pharmacy and Food Sciences, University of Valencia, Av. Vicente Andrés Estellés s/n, 46100 Burjassot, Valencia, Spain

**Keywords:** antiproliferative activity, colorectal cancer, fermentation, food matrix, plant-based beverages

## Abstract

Fermentation represents a sustainable biotechnological approach for enhancing bioactive properties of plant-based foods, yet its anticancer effects remain underexplored. We evaluated the antiproliferative activity of fermented (with commercial probiotic lactic acid bacteria consortium) and unfermented plant-based beverages derived from tiger nut, carob, and rice using an in vitro model. Following INFOGEST 2.0 gastrointestinal digestion, bioaccessible fractions were applied to Caco-2 colorectal adenocarcinoma cells at 1:15 *v*/*v* dilution for 24 h. Analyses included cell viability, apoptosis detection, cell cycle distribution, reactive oxygen species production, glutathione content, mitochondrial membrane potential, and intracellular calcium levels. Fermented tiger nut achieved superior (*p* < 0.05) cytotoxicity compared to unfermented counterpart (39.6% vs. 77.4% cell viability) through dual mechanisms: depleting cellular antioxidant defenses (glutathione reduced to 55.9%) while inducing oxidative stress (180.3% ROS overproduction). This evoked irreversible apoptosis (76.9% early apoptosis) and extensive DNA fragmentation (84.8% SubG_1_ population) via calcium-independent pathways. Fermented carob operated through cytostatic mechanisms, inducing G_0_/G_1_ cell cycle arrest (74.7% vs. 44.2% in blank digestion cells) without oxidative stress. Fermentation reduced (*p* < 0.05) rice beverage antiproliferative activity (90.2% vs. 71.9% unfermented beverage cell viability). These findings establish lactic acid fermentation as effective for developing plant-based beverages with anticancer mechanisms, offering dietary strategies for colorectal cancer prevention.

## 1. Introduction

In recent years, plant-based beverages have gained growing popularity due to increasing consumer awareness of health and sustainability. Among these, formulations based on rice (*Oryza sativa*), tiger nut (*Cyperus esculentus*) and carob (*Ceratonia siliqua*) represent promising alternatives to traditional dairy products owing to their rich profiles of bioactive compounds, low allergenicity and favorable sensory properties [1]. The presence of functional constituents, particularly phenolic compounds, enhances the appeal of these plant matrices not only from a nutritional standpoint but also due to their potential health benefits, as these bioactives have been shown to exhibit antioxidant, anti-inflammatory and antiproliferative properties [2].

Fermentation is a well-established strategy for enhancing the nutritional and functional attributes of plant-based foods and beverages [3]. Through the metabolic activity of lactic acid bacteria (LAB) and other microbial consortia, fermentation can increase the concentration of bioactive compounds and modulate sensory characteristics. Notably, microbial fermentation may facilitate the release and/or biotransformation of phenolics and other phytochemicals into more bioactive forms, thereby influencing their physiological effects in the human body. In particular, fermentation of rice, tiger nut and carob beverages using the commercial LAB consortium Danisco^®^ VEGE061, composed of strains belonging to the genera *Streptococcus*, *Lactobacillus* and *Bifidobacterium*, has been shown to enhance antioxidant capacity and modulate metabolite profiles in ways that suggest improved functional potential [4]. Despite the previously noted enhancement in the bioactive profile of plant-based beverages following fermentation, it remains unclear whether these compositional and antioxidant improvements translate into increased biological activity. This highlights the need for a more direct evaluation of their functional effects, especially given that test-tube antioxidant capacity or phytochemical content do not always correlate with the biological responses observed in cellular models [5]. Recent studies have begun to address this question by comparing fermented and unfermented plant beverages. For instance, Hashemi et al. [6] reported that peach (*Prunus persica*) juice fermented with *Lactobacillus acidophilus* and *Limosilactobacillus fermentum* exhibited superior anti-inflammatory (5-lipoxygenase inhibitory activity, 5–6-fold change), antioxidant (O_2_—radical scavenging, 4–5-fold change) and anti-adhesion properties against *Shigella flexneri* (18–23%) in Caco-2 intestinal epithelial cells, compared to its unfermented counterpart. Similarly, Kim et al. [7] demonstrated that fermentation of kiwi (*Actinidia deliciosa*) juice with *Lactiplantibacillus plantarum* not only elevated total polyphenols (8%) and flavonoids (31%) but also significantly enhanced antioxidant (DPPH (231%) and ABTS+ (13%) radical scavenging activity) and antiproliferative effects against cancer cells, including murine melanoma (B16) (7%), human colorectal adenocarcinoma (HT-29) (27%), human prostate cancer (LNCaP) (4%) and human triple-negative breast cancer (MDA-MB-231) (32%). However, these findings stem from a limited number of studies, and no such functional assessments have yet been reported for fermented beverages based on rice, tiger nut and carob.

In this approach to evaluating the biological activity of functional beverages, it is essential to consider the complex digestive processes that modulate the actual bioactivity of ingested compounds. The INFOGEST 2.0 standardized static in vitro digestion method has emerged as a robust tool for simulating the gastrointestinal environment and assessing the bioaccessibility of nutrients and bioactive compounds [8]. By mimicking oral, gastric and intestinal phases, this model allows for the isolation of the bioaccessible fraction (BF)—i.e., the pool of compounds that becomes available for intestinal absorption—and thus better predicts the in vivo behavior of functional foods. Applying this protocol to both fermented and unfermented plant-based beverages prior to their evaluation in cellular models enables a more accurate simulation of in vivo gastrointestinal conditions [9]. This, in turn, provides a clearer understanding of whether fermentation genuinely enhances the bioactive properties of these beverages.

Among the various biological properties attributed to plant-based functional foods, their potential antiproliferative activity is receiving increasing attention [10]. This is particularly relevant in the case of colorectal cancer (CRC), which remains a leading cause of cancer-related morbidity and mortality worldwide [11]. A commonly used in vitro model for such studies is the human Caco-2 cell line, derived from colorectal adenocarcinoma. Although widely recognized for its use in modeling the intestinal barrier and studying nutrient absorption, the cancerous origin of Caco-2 cells also makes them well-suited for evaluating the cytotoxic and cytostatic effects of bioactive compounds on intestinal tumor cells [12].

## 2. Materials and Methods

### 2.1. Reagents

pH measurement strips (Mquant) were sourced from Merck KGaA (Darmstadt, Germany), alongside potassium dibasic phosphate (K_2_HPO_4_) and sodium dibasic phosphate (Na_2_HPO_4_) from Merck (Darmstadt, Germany). A comprehensive range of biochemical reagents was procured from Merck Life Science S.L.U. (Madrid, Spain), encompassing human salivary α-amylase (E.C 3.2.1.1), ammonium carbonate, ammonium chloride, anhydrous sodium sulfate, bovine bile, calcium chloride dihydrate, hydrochloric acid (37% purity), magnesium chloride hexahydrate, methanol, pancreatin extract from porcine pancreas, porcine pepsin (E.C 3.4.23.1), potassium chloride, potassium dihydrogen phosphate, potassium hydroxide, sodium chloride, and sodium hydroxide. Specialized digestive enzymes included rabbit gastric extract (RGE) acquired from Lipolytech (Marseille, France), while sodium bicarbonate was sourced from Panreac (Barcelona, Spain). Cell culture media and supplements were provided by multiple vendors: Dulbecco’s Modified Eagle Medium (DMEM) + GlutaMAX™ (4.5 g/L glucose) was acquired from Invitrogen™ (Thermo Fisher Scientific, Eugene, OR, USA), whereas Gibco™ (Scotland, UK) supplied MEM Non-Essential Amino Acids (MEM NEAA) solution (100×), HEPES buffer solution (1 M), antibiotic solution (10,000 U/mL penicillin and 10,000 μg/mL streptomycin), antimycotic solution (250 μg/mL amphotericin B), fetal bovine serum (FBS), PBS pH 7.4 (1×), and trypsin-EDTA (ethylenediaminetetraacetic acid) solution (2.5 g/L trypsin and 0.2 g/L EDTA). Analytical compounds for cytotoxicity and flow cytometry assays were acquired from various sources: dimethyl sulfoxide (DMSO), 3-(4,5-dimethylthiazol-2-yl)-2,5-diphenyl-tetrazolium bromide (MTT), 5-fluorouracil (5-FU), propidium iodide (PI), and 2′,7′-dichlorofluorescein diacetate (DCFDA) were all sourced from Merck LifeScience S.L.U. (Madrid, Spain). The FITC (fluorescein isothiocyanate) Annexin V apoptosis detection kit I was provided by BD Biosciences (San Jose, CA, USA). Additional fluorescent probes included 5-chloromethylfluorescein acetate (Green CMFDA) from Abcam (Cambridge, MA, USA), 3,3-dihexyloxacarboxycyanine iodide (DiOC_6_) from Invitrogen™ (Thermo Fisher Scientific, Eugene, OR, USA), and Fluo-3/acetoxymethyl (FLUO 3/AM) from Santa Cruz Biotechnology (Heidelberg, Germany). Finally, ethanol (96%) was supplied by Scharlau (Barcelona, Spain), and deionized water (resistivity 18.2 MΩ cm) was generated using a Milli-Q water purification system (Millipore™, Bedford, MA, USA) 

### 2.2. Preparation of Fermented Beverages

The fermentation process utilized the commercial probiotic LAB consortium VEGE061 (Danisco, Denmark), which contains a mixture of beneficial bacterial strains including *Streptococcus thermophilus*, *Lactobacillus delbrueckii* subsp. *bulgaricus*, *Lactobacillus acidophilus* NCFM^®^, *Bifidobacterium animalis* subsp. *lactis* HN019^®^, and *Lactobacillus paracasei* (*Lacticaseibacillus casei*).

The experimental protocol consisted of distributing 800 mL of each plant beverage in 1 L glass containers. Following manufacturer specifications, the starter culture VEGE061 was added to initiate the fermentation process. All samples underwent fermentation at a constant temperature of 37 °C until the desired acidity level (pH 4.0–4.5) was achieved. The time required to reach this pH range varied depending on the plant matrix: tiger nut beverage required 24 h, rice beverage needed 48 h, while carob beverage demanded the longest fermentation period of 72 h. The proximate composition of beverages is also mentioned in previous articles [4].

### 2.3. Simulation of Gastrointestinal Digestion

After the corresponding fermentation, the beverages were subjected to a standardized three-phase simulated gastrointestinal digestion following the INFOGEST 2.0 protocol [8]. Three simulated digestive fluids were prepared: simulated salivary fluid (SSF), simulated gastric fluid (SGF), and simulated intestinal fluid (SIF), with pH adjusted to 7.0 for SSF and SIF, and to 3.0 for SGF using HCl (6 M) or NaOH (6 M) as needed. Enzyme solutions were prepared separately as concentrated stocks according to specific activity calculations.

The digestion process began with the oral phase, where 5 g of each sample (or water for blank of digestion) was mixed with 3.5 mL of simulated salivary fluid (pH 7.0), 0.5 mL of α-amylase solution to achieve a final concentration of 75 U/mL, 25 μL of calcium chloride solution (0.3 M), and 3.975 mL of Milli-Q water to reach a final volume of 10 mL. This mixture underwent gentle agitation in a temperature-controlled shaking bath at 37 °C and 95 rpm for 2 min.

Following the oral phase, gastric digestion was initiated by adding 7.5 mL of SGF (pH 3.0), 0.98 mL of RGE solution providing a final concentration of 60 U lipase/mL, 0.62 mL of pepsin solution to achieve a final concentration of 2000 U/mL, 5 μL of calcium chloride solution (0.3 M), and 895 μL of Milli-Q water to achieve a final volume of 20 mL. The pH was adjusted to 3.0 using HCl (6 M), and the gastric mixture was incubated for 2 h under controlled conditions at 37 °C and 95 rpm.

The final intestinal phase commenced with the addition of 11.0 mL of simulated intestinal fluid (pH 7.0), 5.0 mL of pancreatin solution to achieve a final concentration of 100 U/mL, 2.5 mL of bile salts solution to have a final concentration of 10 mM, 40 μL of calcium chloride solution (0.3 M), and Milli-Q water to bring the total volume to 40 mL. The pH was adjusted to 7.0 using NaOH (6 M), and the mixture was incubated for 2 h at 37 °C and 95 rpm. After completing the digestion sequence, the BF was isolated through centrifugation at 3100× *g* for 90 min at 4 °C.

The BFs obtained after in vitro digestion have been previously characterized by our research group in terms of their bioactive compound content and antioxidant capacity (unpublished results). Table 1 presents the concentration of total soluble polyphenol content (TSP) and total antioxidant capacity (measured by ORAC and TEAC assays) in the BFs used for the antiproliferative evaluation in Caco-2 cells. These previously obtained data are presented here to provide context for understanding the potential biological activity and the observed antiproliferative effects of the beverages.

### 2.4. In Vitro Cell Culture Experimental Design

The human colorectal adenocarcinoma cell line (Caco-2) and normal human colon fibroblasts (CCD-18Co) were both acquired from the American Type Culture Collection repository (HTB-37 and CRL-1459, respectively, Rockville, Maryland). These cell lines were propagated in Corning™ Falcon™ tissue culture flasks (75 cm^2^) containing DMEM enriched with FBS (10% *v*/*v*), MEM NEAA (1% *v*/*v*), antibiotic solution (1% *v*/*v*), antifungal solution (0.2% *v*/*v*), and HEPES buffer (1% *v*/*v*). Cells were maintained at 37 °C, with 95% relative humidity and a 5% CO_2_ atmosphere. For experimental protocols, Caco-2 (passage 25–35) and CCD-18Co cells (passages 42–45) were seeded at specific densities depending on the assay: 25,000 cells per well in 96-well microplates for MTT viability assays, and 100,000 cells per well in 24-well culture plates for flow cytometry analyses. Following a 24 h attachment period, The BFs were tested at various dilutions (1:5, 1:10, 1:12.5, 1:15, and 1:20, *v*/*v*) using the MTT assay on Caco-2 cells to determine the optimal concentration. Subsequently, the selected optimal dilution (1:15 *v*/*v*) was tested on both Caco-2 and CCD-18Co cell lines to assess selective cytotoxicity. Untreated cells served as negative controls, while 5-fluorouracil (25 μM) was used as a positive cytotoxicity reference [13]. All treatments were performed for 24 h under standard culture conditions (37 °C, 95% relative humidity, 5% CO_2_ atmosphere).

### 2.5. Cytotoxicity Assay

Cell viability was assessed using the MTT colorimetric method [14]. This technique measures metabolic activity as a surrogate marker for cell viability, based on the enzymatic conversion of the yellow tetrazolium compound to purple formazan crystals by metabolically active cells. Following the 24 h treatment period, culture media were aspirated from the wells, and fresh MTT solution (0.5 mg/mL prepared in PBS) was added (100 μL per well). The plates were incubated at 37 °C with 95% relative humidity and 5% CO_2_ atmosphere for 4 h. Subsequently, the MTT-containing solution was carefully removed, and the resulting formazan precipitates were dissolved by adding 100 μL of DMSO to each well. Quantification of the colored reaction product was performed using a Multiskan EX spectrophotometric plate reader (Thermo Scientific, Waltham, MA, USA) measuring absorbance at 570 nm, with background correction at 690 nm. Cell viability results were calculated as a percentage relative to blank digestion.

### 2.6. Cell Death

Apoptotic cell death was evaluated through dual fluorescent labeling using the FITC-Annexin V apoptosis detection kit I. This approach simultaneously identifies externalized phosphatidylserine residues via Annexin V-FITC binding and compromised nuclear integrity through propidium iodide (PI) incorporation [15]. Following treatment exposure, cellular populations were detached using 400 μL trypsin-EDTA solution and subsequently collected in 5 mL round-bottom flow cytometry tubes (Corning™ Falcon™). Samples underwent centrifugation at 450× *g* for 5 min at 25 °C using an Eppendorf^®^ centrifuge 5810R. The resulting pellets were resuspended by adding 100 μL binding buffer along with 5 μL of Annexin and 5 μL of PI solutions. Staining proceeded under light-protected conditions for 15 min at room temperature. Subsequently, samples were diluted with an additional 400 μL of binding buffer prior to flow cytometric evaluation. Analysis was performed using a FACS Verse system (BD Biosciences) with fluorescence detection parameters set at λ_exc_ = 488 nm and λ_em_ = 527/32 nm for FITC-Annexin V and λ_exc_ = 488 nm and λ_em_ = 586/42 nm for PI, acquiring a minimum of 10,000 events per sample. To ensure data quality, cell suspensions were passed through 50 μm filters (CellTrics^®^) for debris elimination. Background fluorescence was established using unstained control populations, while compensation for spectral interference was achieved through single-fluorochrome stained samples.

### 2.7. Analysis of Cell Cycle Progression

The distribution of cells across different phases of the cell cycle was determined by quantitative DNA content analysis using flow cytometry [15]. This technique employs PI, a fluorescent intercalating agent that binds to nucleic acids in direct proportion to their abundance, enabling precise measurement of cellular DNA content.

Following treatment, cells were harvested and immediately subjected to fixation and permeabilization using a chilled solution of ethanol and PBS (70:30 *v*/*v*, 1 mL) for 30 min at 4 °C. After centrifugation, the resulting cell pellet was resuspended in 400 μL of fresh PBS. To ensure DNA-specific staining by eliminating RNA interference, the suspension was treated with 50 μL of RNase A solution (40 μg/mL in PBS) simultaneously with 50 μL of PI staining solution (100 μg/mL in PBS).

The preparation was then incubated at 37 °C under standard culture conditions (95% relative humidity, 5% CO_2_ atmosphere) for 30 min to allow complete enzymatic digestion of RNA and thorough DNA staining. Prior to analysis, the cell suspension was passed through a 50 μm filter (CellTrics^®^) to remove cellular aggregates. Fluorescence intensity measurements were performed using flow cytometry (FACS Verse, BD Biosciences) with excitation at 488 nm and emission detection at 527/32 nm. A minimum of 10,000 individual cellular events were recorded for each experimental condition.

### 2.8. Cellular Levels of Reactive Oxygen Species

Intracellular reactive oxygen species (ROS) levels were measured to assess oxidative stress induction by the BF treatments [15]. The method employs DCFD, which in the presence of ROS is oxidized to fluorescent dichlorofluorescein, enabling quantification by flow cytometry.

After harvesting cells following treatment, they were resuspended in 1 mL of 10 μM DCFDA solution in a cytometry tube and incubated in darkness for 30 min at 37 °C with 95% relative humidity and 5% CO_2_. Following incubation, the tubes were centrifuged (450× *g*, 5 min, 25 °C), and the pellet was resuspended in 300 μL of PBS.

Fluorescent intensity was analyzed using flow cytometry (FACS Verse, BD Biosciences) with excitation at 488 nm and emission detection at 527/32 nm. Data were collected from at least 10,000 events per sample, after subtracting cellular autofluorescence. Results were expressed as percentage relative to the digestion blank control.

### 2.9. Intracellular Glutathione (GSH) Determination

Intracellular GSH content was measured using CMFDA, a cell-permeable probe that selectively binds to non-protein thiol groups, predominantly GSH [15]. After the 24 h treatment period, 500 μL of cell suspension was combined with CMFDA to reach a final concentration of 1 μM. This mixture was incubated for 40 min under standard culture conditions (37 °C, 5% CO_2_, 95% relative humidity). Following incubation, the cells were pelleted by centrifugation (450× *g*, 5 min, 25 °C) and resuspended in 300 μL of PBS for immediate analysis. Quantification was performed using flow cytometry (FACS Verse, BD Biosciences) with excitation at 485 nm and emission detection at 530 nm. Data were collected from a minimum of 10,000 individual cellular events per sample. Results were expressed as mean fluorescence intensity relative to the digestion blank control.

### 2.10. Mitochondrial Membrane Potential Changes (ΔΨm)

Mitochondrial membrane potential was evaluated using the lipophilic fluorescent probe DiOC_6_ [15]. Both floating and trypsinized cells were collected in cytometry tubes and incubated with DiOC_6_ solution (10 μM) for 15 min in darkness at room temperature.

After incubation, cells were centrifuged (450× *g*, 5 min, 25 °C), and the pellet was resuspended in 300 μL of PBS. Fluorescence was measured by flow cytometry (FACS Verse, BD Biosciences) with excitation at 485 nm and emission detection at 499 nm. Data were collected from a minimum of 10,000 cellular events per sample.

Results were expressed as percentage of depolarization relative to the digestion blank control, with decreased fluorescence indicating mitochondrial membrane depolarization.

### 2.11. Cytosolic Calcium (Ca^2+^) Content

Intracellular Ca^2+^ concentrations were measured using the calcium-sensitive fluorescent indicator Fluo-3/AM [15]. Both suspended and adherent cells were collected, centrifuged and resuspended in 500 μL of PBS. Under dark conditions, 10 μL of Fluo-3/AM solution (100 μM) was added, followed by 40 min of incubation. After centrifugation, cells were resuspended in 300 μL of PBS. Calcium-bound Fluo-3 fluorescence was detected by flow cytometry (excitation: λ_exc_ 506 nm, emission: λ_em_ 526 nm) and quantified as fluorescence intensity in a minimum of 10,000 events per sample; the result was expressed as a percentage against the digestion blank.

### 2.12. Statistical Analysis

Data analysis was performed using GraphPad Prism version 9.0 (GraphPad Software, San Diego, CA, USA). Results are expressed as mean ± standard deviation from at least three independent experiments, each performed in triplicate.

Statistical significance was evaluated using Student’s *t*-test when comparing each treatment to the digestion blank control. Additional paired *t*-test comparisons were performed between fermented and unfermented beverages of the same matrix (i.e., tiger nut vs. fermented tiger nut, carob vs. fermented carob, and rice vs. fermented rice).

All statistical tests and differences were considered statistically significant at *p* < 0.05.

## 3. Results

### 3.1. Effect of BF on Cell Viability in Caco-2 Cells

**Figure 1:**
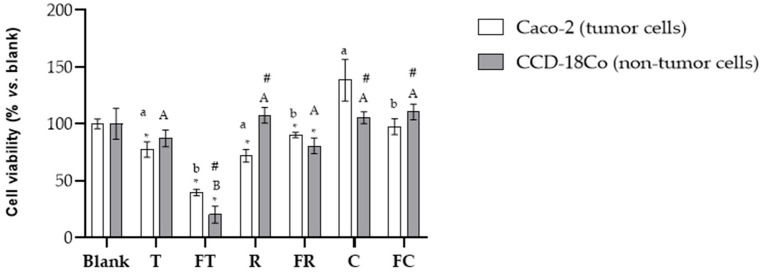
Evaluation of the cytotoxic effects of BFs from both fermented and unfermented plant-based beverages on colorectal adenocarcinoma Caco-2 cells and normal colon fibroblast CCD-18Co cells. Samples evaluated include tiger nut beverage (T), fermented tiger nut beverage (FT), carob beverage (C), fermented carob beverage (FC), rice beverage (R), and fermented rice beverage (FR). * Indicates statistical significant differences compared to digestion blank and different letters indicate statistical significant differences between fermented and unfermented samples: lowercase letters for Caco-2 cells and uppercase letters for CCD-18Co cells, # indicate difference between tumor and non- tumor cells (Student’s *t*-test; *p* < 0.05).

The BFs were tested at the aforementioned dilutions (Section 2.4) using the MTT assay on Caco-2 cells (data shown in Supplementary Material Figures S1–S3). The 1:15 *v*/*v* dilution was selected for all subsequent analyses as it demonstrated the optimal selective effect, reducing tumoral Caco-2 cell viability by approximately 50% while having, in general, minimal cytotoxic impact on the non-tumoral CCD-18Co cells. This selective antiproliferative effect against cancer cells, with preservation of healthy colon cell viability, suggests potential for targeted anticancer activity with reduced side effects on normal tissues. The BFs of the beverages exhibited varying degrees of antiproliferative activity against Caco-2 colorectal cancer cells, with effects significantly influenced by both the plant matrix and fermentation process (Figure 1). Tiger nut beverage significantly reduced (*p* < 0.05) the viability of Caco-2 cells to 77.4% compared to the blank digestion control. Fermentation enhanced this antiproliferative effect, with the fermented tiger nut beverage achieving the strongest cytotoxic response among all treatments, reducing cell viability to 39.6% compared to the blank digestion (*p* < 0.05), representing a significant improvement over the unfermented tiger nut beverage (as deduced from the increase in TSP and ORAC, see Table 1). However, fermented tiger nut showed significantly higher cytotoxicity toward normal CCD-18Co cells (30% viability) than toward Caco-2 cancer cells (*p* < 0.05), indicating lack of cancer cell selectivity in the MTT assay.). Unfermented rice beverage demonstrated notable antiproliferative activity, reducing cell viability to 71.9%. However, fermentation resulted in a significant reduction in this beneficial effect, with fermented rice beverage showing diminished cytotoxicity and higher cell viability at 90.2% (*p* < 0.05). Additionally, non-fermented rice beverages showed statistically significant selective cytotoxicity between Caco-2 and CCD-18Co cells. This represents an 18.3% decrease in antiproliferative potential following fermentation, suggesting that the fermentation process may have degraded key bioactive compounds responsible for the anticancer activity in rice (see a trend to decrease in TSP and ORAC, Table 1). On the other hand, carob beverages showed no antiproliferative activity, with unfermented and fermented formulations resulting in cell viabilities of 138.4% and 97.4%, respectively, with no significant differences. Both values remained close to or above digestion blank levels, indicating minimal anticancer efficacy under the tested conditions. Importantly, fermented carob demonstrated statistically significant differences in cell viability between Caco-2 and CCD-18Co cells, indicating selective anticancer activity.

### 3.2. Effect of Beverage BFs on Programmed Death Mechanisms

**Figure 2:**
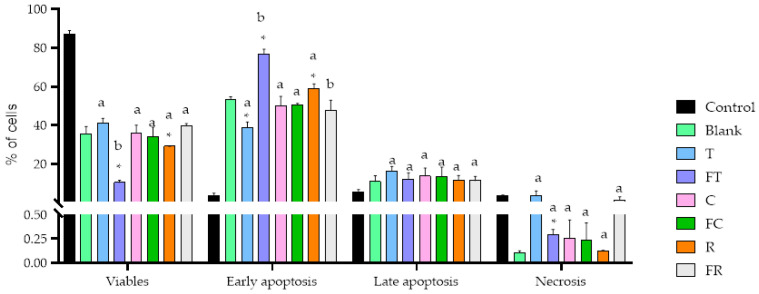
Effects of BFs of fermented and unfermented plant-based beverages on cell death patterns in Caco-2 cells. Samples evaluated include tiger nut beverage (T), fermented tiger nut beverage (FT), carob beverage (C), fermented carob beverage (FC), rice beverage (R), and fermented rice beverage (FR). * Indicates statistical significant differences compared to blank digestion and different letters indicate statistical significant differences between fermented and unfermented samples (Student’s *t*-test; *p* < 0.05).

Cell death analysis revealed distinct patterns of apoptosis induction across treatments, with notable differences in the mechanisms and intensity of cell death between fermented and unfermented beverages (Figure 2).

Fermented tiger nut beverage exhibited the most potent pro-apoptotic effect among all treatments, dramatically reducing viable cell populations to just 10.5% while inducing early apoptosis in 76.9% of cells (*p* < 0.05). This represents a significant enhancement compared to its unfermented counterpart (*p* < 0.05), demonstrating that fermentation significantly amplifies the pro-apoptotic potential of tiger nut beverage. This pronounced apoptotic response correlates directly with the significant reduction in cell viability observed in the MTT assay, confirming that the cytotoxic effect is primarily mediated through programmed cell death rather than necrosis. Additionally, fermented tiger nut beverage showed a small but statistically significant increase in necrosis compared to the digestion blank control, indicating activation of multiple cell death pathways. Unfermented rice beverage demonstrated significant (*p* < 0.05) apoptotic activity, with 59.1% of cells undergoing early apoptosis. This finding provides mechanistic insight into the moderate antiproliferative effect observed for rice beverage in the viability assay confirming that rice-derived bioactive compounds can effectively trigger apoptotic cell death in colorectal cancer cells. The remaining treatments showed no induction of apoptotic cell death, which is consistent with their limited impact on cell viability.

### 3.3. Effect of BFs Beverages on Cell Cycle Distribution in Caco-2 Cells

**Figure 3:**
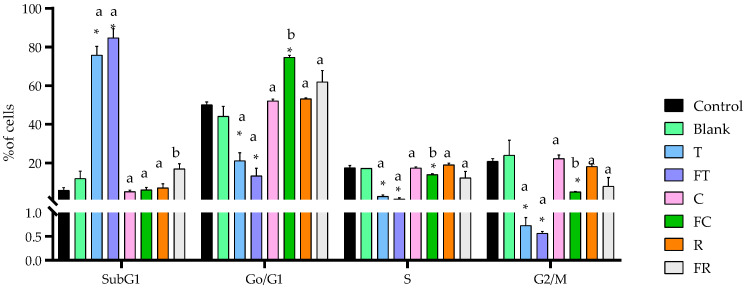
Effects of BFs of fermented and unfermented plant-based beverages on cell cycle distribution in Caco-2 cells. Samples evaluated include tiger nut beverage (T), fermented tiger nut beverage (FT), carob beverage (C), fermented carob beverage (FC), rice beverage (R), and fermented rice beverage (FR). * Indicates statistical significant differences compared to blank digestion control, and different letters indicate statistical significant differences between fermented and unfermented samples (Student’s *t*-test; *p* < 0.05).

Cell cycle analysis revealed distinct patterns of cycle modulation that varied significantly between treatments, providing mechanistic insights into the different antiproliferative strategies employed by each beverage matrix (Figure 3).

Tiger nut beverages, both unfermented and fermented, caused a dramatic accumulation of cells in the SubG_1_ population, reaching 75.8% and 84.8%, respectively, compared to the digestion blank control (12% in subG_1_ phase *p* < 0.05). This pronounced elevation in SubG_1_ cells, which represents DNA fragmentation characteristic of apoptotic processes, directly correlates with the high levels of early apoptosis observed in the previous analysis (76.9% for fermented tiger nut). Additionally, both tiger nut formulations showed significant concomitant reductions in S and G_2_/M phases, indicating complete inhibition of DNA synthesis and cell division processes. Fermented carob beverage induced a significantly different response, causing the most pronounced cell cycle arrest in the G_0_/G_1_ phase (74.7%) compared to its unfermented counterpart (52.1% *p* < 0.05), being higher (*p* < 0.05) than the values for blank of digestion control (44.2%). This substantial increase in G_0_/G_1_ arrest suggests a cytostatic rather than cytotoxic mechanism, where cells are prevented from progressing through the cell cycle without immediate induction of cell death. This finding explains the limited cytotoxic effects observed in the viability assays while confirming antiproliferative activity through cell cycle inhibition. Rice beverages exhibited moderate cell cycle effects, with both unfermented and fermented formulations showing G_0_/G_1_ arrest (53.2% and 61.9%, respectively) with no significant differences (*p* > 0.05) between fermentation treatments compared to the blank (44.2%). This moderate trend to arrest in this cell cycle phase may contribute to the antiproliferative activity observed with rice beverage, although the effect was less pronounced than that seen with carob fermentation.

### 3.4. Effect of BFs Beverages on Intracellular ROS Levels in Caco-2 Cells

**Figure 4:**
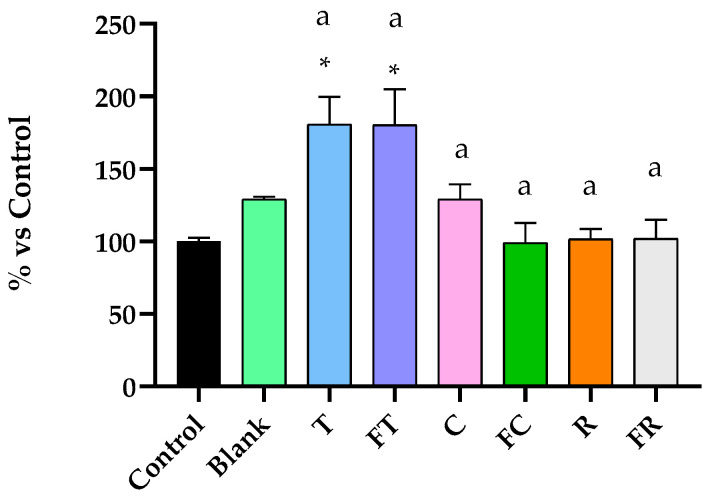
Effects of BFs of fermented and unfermented plant-based beverages on intracellular reactive oxygen species (ROS) levels in Caco-2 cells. Results are expressed as percentage relative to control cells. Samples evaluated include tiger nut beverage (T), fermented tiger nut beverage (FT), carob beverage (C), fermented carob beverage (FC), rice beverage (R), and fermented rice beverage (FR). * Indicates statistical significant differences compared to digestion blank, and different letters indicate statistical significant differences between fermented and un-fermented samples (Student’s *t*-test; *p* < 0.05).

The evaluation of intracellular ROS production revealed distinct oxidative stress patterns that strongly correlate with the antiproliferative mechanisms observed in previous analyses (Figure 4). 

Tiger nut beverages demonstrated the unique and most remarkable pro-oxidant effects among all treatments, with both unfermented and fermented variants achieving similarly elevated ROS levels at 180.8% and 180.3% of the digestion blank, respectively. These substantial ROS elevations were statistically significant and represent the highest oxidative stress induction observed in the study. The similarity in ROS production between fermented and unfermented tiger nut formulations suggests that the enhanced cytotoxicity of the fermented variant is likely due to qualitative changes in bioactive compounds, specifically fermentation-induced enrichment of ferulic acid and other phenolic metabolites [4], rather than increased ROS generation alone. Carob beverages exhibited contrasting unsignificant trend in ROS responses depending on fermentation status. Unfermented carob induced a moderate but unsignificant increase in ROS production (129.2% vs. control), while fermented carob showed no significant elevation (99.0% vs. control). This fermentation-dependent reduction in ROS generation aligns with the cytostatic rather than cytotoxic profile observed for fermented carob, where cell cycle arrest occurs without significant oxidative stress or apoptosis induction.

Rice beverages produced minimal effects on cellular redox status, with both unfermented and fermented formulations showing only slight, unsignificant changes in ROS levels (101.6% and 102.0%, respectively). These minimal oxidative changes suggest that the moderate antiproliferative effects observed with rice beverages operate through ROS-independent mechanisms. The ROS production patterns establish a clear mechanistic framework: tiger nut beverages employ an oxidative stress-mediated apoptotic pathway, while carob beverages (particularly fermented) achieve minimal antiproliferative effects through redox-independent cell cycle modulation. This differential approach provides valuable insights into the diverse mechanisms by which plant-based bioactive compounds can target cancer cell proliferation.

### 3.5. Effect of BFs Beverages on Intracellular GSH Content in Caco-2 Cells

**Figure 5:**
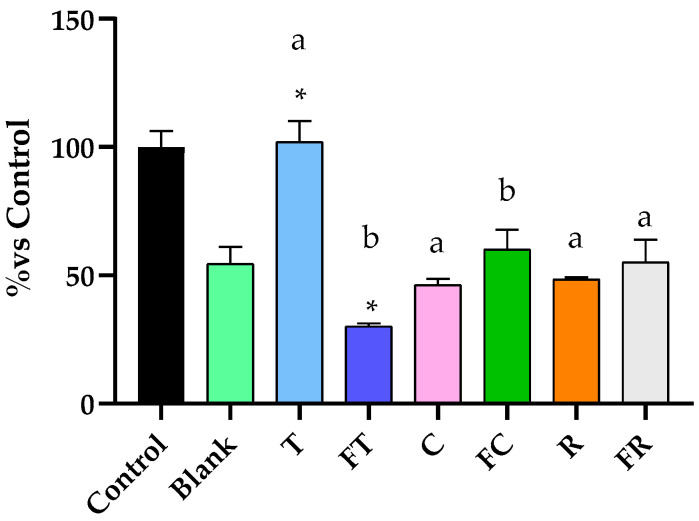
Effects of BFs of fermented and unfermented plant-based beverages on intracellular glutathione (GSH) levels in Caco-2 cells. Results are expressed as percentage relative to control cells. Samples evaluated include tiger nut beverage (T), fermented tiger nut beverage (FT), carob beverage (C), fermented carob beverage (FC), rice beverage (R), and fermented rice beverage (FR). * Indicates statistical significant differences compared to digestion blank and different letters indicate statistical significant differences between fermented and unfermented samples (Student’s *t*-test; *p* < 0.05).

The GSH content revealed critical insights into cellular antioxidant defense mechanisms and their relationship with the oxidative stress patterns observed in the previous analysis (Figure 5).

It should be noted that the digestion blank control (containing digestive enzymes and bile salts from the INFOGEST 2.0 protocol) showed inherent effects on cellular GSH levels (54.41 ± 6.64% relative to untreated control cells). The bile salts and digestive enzymes can influence cellular antioxidant systems. However, all results were expressed relative to this digestion blank control, ensuring that the observed differences between treatments reflect the specific effects of the plant-based beverages rather than artifacts from the digestion process. The most striking finding was the differential impact of tiger nut beverages on GSH levels. While unfermented tiger nut beverage maintained GSH at levels comparable to the control cells (102.1%), fermented tiger nut beverage caused a severe and significant depletion of GSH levels compared to the digestion blank, reducing them to 30.2% (*p* < 0.05). This profound difference provides a mechanistic explanation for the enhanced cytotoxicity of fermented tiger nut despite similar ROS production levels (180.8% vs. 180.3%). The remaining beverages demonstrated more uniform GSH modulation patterns with no significant differences versus blank of digestion. This fact reflects mild to moderate oxidative stress that does not overwhelm cellular defenses, consistent with the limited ROS overproduction observed with these treatments.

### 3.6. Effect of BFs Beverages on Mitochondrial Membrane Potential Changes (ΔΨm)

**Figure 6:**
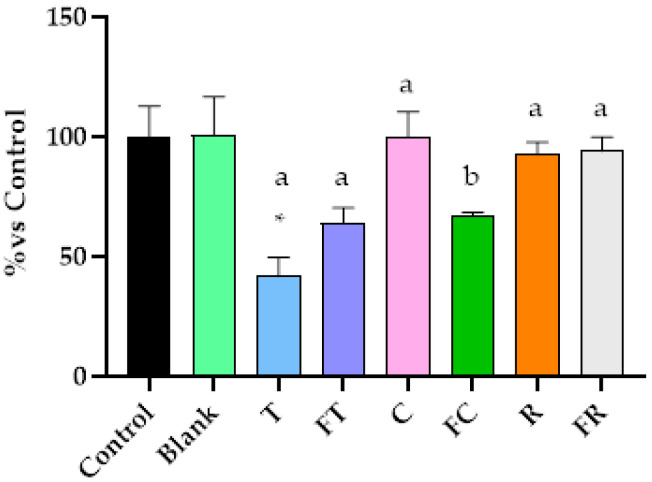
Effects of BFs of fermented and unfermented plant-based beverages on mitochondrial membrane potential changes (ΔΨm) in Caco-2 cells. Results are expressed as percentage relative to control cells. Lower values indicate greater mitochondrial membrane depolarization. Samples evaluated include tiger nut beverage (T), fermented tiger nut beverage (FT), carob beverage (C), fermented carob beverage (FC), rice beverage (R), and fermented rice beverage (FR). * Indicates statistical significant differences compared to blank of digestion and different letters indicate statistical significant differences between fermented and unfermented samples (Student’s *t*-test; *p* < 0.05).

Assessment of mitochondrial integrity revealed selective alterations in membrane potential in specific treatment groups (Figure 6). The unfermented tiger nut beverage induced the most pronounced mitochondrial membrane depolarization, with values reduced (*p* < 0.05) to only 41.5% of the digestion blank. This marked disruption of mitochondrial function aligns with the previously observed pro-apoptotic effects and suggests activation of the intrinsic apoptotic pathway. FT also caused a substantial non-significant (*p* > 0.05) mitochondrial depolarization (63.7% of blank of digestion), though less severe than its unfermented counterpart (no significant differences between treatments). FC demonstrated moderate but non-significant (*p* > 0.05) mitochondrial membrane depolarization (67.1%) versus blank of digestion, which is particularly interesting given that it did not induce significant ROS production or GSH depletion. This suggests that FC may influence mitochondrial function through mechanisms independent of oxidative stress. In contrast, unfermented carob (C) and both rice beverages (R and RF) maintained mitochondrial membrane potential at levels comparable to the digestion blank (99.6%, 92.3%, and 93.7%, respectively). These findings establish a clear connection between the cytotoxic effects of tiger nut beverages and mitochondrial dysfunction, with the severity of membrane potential disruption correlating well with their pro-apoptotic capacity. The differential responses observed between fermented and unfermented variants highlight the complexity of bioactive compound interactions with cellular metabolic pathways.

### 3.7. Effect of BFs Beverages on Cytosolic Calcium Content

**Figure 7:**
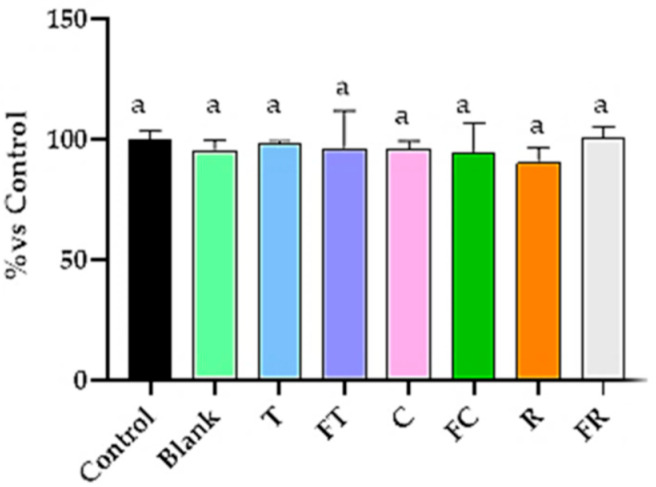
Effects of BFs of fermented and unfermented plant-based beverages on intracellular calcium levels in Caco-2 cells. Results are expressed as percentage relative to control cells. Samples evaluated include tiger nut beverage (T), fermented tiger nut beverage (FT), carob beverage (C), fermented carob beverage (FC), rice beverage (R), and fermented rice beverage (FR). Different letters indicate statistical significant differences between fermented and unfermented samples (Student’s *t*-test; *p* < 0.05).

The analysis of cytosolic calcium concentrations revealed no significant alterations across any of the tested beverages, with all treatments maintaining calcium levels comparable to the control (Figure 7).

## 4. Discussion

Colorectal cancer (CRC) remains a leading cause of cancer-related morbidity and mortality worldwide, ranking as the third most diagnosed cancer and the second deadliest [11]. Despite advances in screening, surgical techniques and pharmacological therapies, CRC continues to pose a significant public health challenge due to its often late-stage diagnosis and high recurrence rates. One of the major obstacles in effective treatment is the resistance of cancer cells to apoptosis—a form of programmed cell death essential for eliminating abnormal cells—and their capacity for uncontrolled proliferation [16]. These characteristics are well-established hallmarks of CRC and contribute to both tumor progression and therapy resistance. Consequently, there is growing interest in identifying complementary strategies that can augment the efficacy of conventional treatments [17]. Among these, dietary interventions and the use of bioactive compounds derived from food have emerged as promising avenues for modulating cancer cell behavior, improving patient outcomes and potentially reducing treatment-related side effects.

In this context, plant-based beverages have emerged as promising candidates for nutritional interventions, owing to their naturally high content of phytochemicals, particularly polyphenols, which possess well-documented antiproliferative properties [18]. These compounds can modulate signaling pathways involved in carcinogenesis and inhibit the proliferation of malignant cells, thereby contributing to cancer prevention and complementary management strategies. Notably, the health-promoting potential of these beverages can be significantly enhanced through fermentation, a sustainable and cost-effective biotechnological process [3]. Fermentation not only improves the sensory and nutritional qualities of plant matrices but also alters their biochemical composition in ways that increase the bioavailability and bioaccessibility of beneficial metabolites. Moreover, microbial fermentation can lead to the production of novel bioactive compounds that are not present in the raw materials, some of which have demonstrated promising anticancer effects in pre-clinical models [19]. As such, fermented plant-based beverages represent an innovative and multifunctional dietary approach with the potential to support conventional CRC therapies by targeting multiple mechanisms involved in tumor development and progression. In this sense, it has been demonstrated that fermentation of kiwi juice with *Lactobacillus plantarum* significantly enhanced the juice’s ability to inhibit cancer cell proliferation, showing notable antiproliferative effects against various cancer cell lines, including HT-29 human colorectal adenocarcinoma cells [7]. These findings underscore the potential of fermented plant-based products to exert targeted biological activity against tumor cells, particularly in the context of CRC.

Despite these promising results, studies evaluating the antiproliferative effects of fermented plant-based beverages remain limited in number. Addressing this gap, the present study evaluated the antiproliferative effects of fermented and unfermented beverages derived from rice, carob and tiger nut on Caco-2 cells, a well-established in vitro model for CRC. Our results demonstrated significant differences in cytotoxicity, apoptosis induction and cell cycle modulation, with fermented tiger nut beverage showing the most pronounced effects, since fermentation intensified the cell viability decrease in this food matrix (Figure 1). The enhanced cytotoxicity observed in the fermented variant was accompanied by a substantial increase in early apoptotic cells (Figure 2), as well as a pronounced elevation in the SubG_1_ cell population (Figure 3), a marker commonly associated with DNA fragmentation and apoptosis. These findings suggest that fermentation amplifies the antiproliferative potential of the tiger nut beverage, reinforcing its potential as a functional food component with chemopreventive properties.

Our findings align with previous studies demonstrating that fermentation with the Danisco^®^ VEGE061 LAB consortium can enhance the bioactive profile of tiger nut beverage, increasing the concentration of compounds such as homovanillic acid, L-leucic acid and particularly ferulic acid [4] (Supplementary Table S1). Ferulic acid has been previously reported to exert notable antiproliferative effects against Caco-2 cells at concentrations ranging from 150 to 1500 μM over exposure periods of 1 to 3 days, by reducing cell proliferation, inducing S-phase cell cycle arrest and modulating the expression of proteins involved in the regulation of cell proliferation [20,21]. Therefore, the elevated total antioxidant capacity (TSP and ORAC values, Table 1) as well as of ferulic acid levels generated during fermentation may partially explain the enhanced apoptotic and cytotoxic effects observed in fermented tiger nut beverage, supporting its potential role in CRC prevention or adjunctive therapy.

Furthermore, the antiproliferative effects of the unfermented and fermented tiger nut beverages were accompanied by remarkable overproduction of ROS in Caco-2 cells (Figure 4). This increase in intracellular ROS is a well-established mechanism by which phytochemicals exert pro-apoptotic effects, disrupting cellular redox homeostasis and triggering signaling pathways involved in programmed cell death [22]. Importantly, this ROS overproduction occurred despite the increased antioxidant capacity observed in test-tube assays (TSP and ORAC values, Table 1), illustrating the well-documented paradoxical behavior of polyphenols [5]. While these compounds act as antioxidants in simple chemical systems, they can become pro-oxidants in cellular environments through interactions with metal ions and metabolic transformation, particularly beneficial for inducing apoptosis in cancer cells [23]. Additionally, the ROS overproduction was paralleled by significant depolarization of the mitochondrial membrane potential (Figure 6), a hallmark event in the intrinsic (mitochondrial) apoptotic pathway [24]. Mitochondrial membrane depolarization compromises mitochondrial integrity, potentially facilitating the release of pro-apoptotic factors such as cytochrome C into the cytosol. This release may, in turn, activate downstream caspase cascades, leading to the execution phase of apoptosis.

Critically, these redox disturbances were accompanied by differential modulation of intracellular GSH levels (Figure 5). While exposure to the unfermented tiger nut beverage increased GSH content up to normal levels (102.1%)—indicating cellular adaptive response to oxidative stress—the fermented tiger nut beverage led to significantly depletion of GSH compared to blank of digestion values. This reduction in GSH suggests significant oxidative stress, likely driven by the presence of fermentation-derived metabolites with known pro-oxidant activity. We hypothesize that this GSH depletion pattern represents a potential therapeutic mechanism employed by the fermented tiger nut beverage: rather than simply inducing cellular damage, it may systematically compromise cellular antioxidant defenses before delivering oxidative stress, potentially creating a mechanism that impairs cellular recovery and promotes apoptotic commitment. This hypothesis may explain an apparent paradox observed in the mitochondrial membrane potential analysis (Figure 6), where unfermented tiger nut beverage induced greater mitochondrial depolarization yet resulted in higher cell survival compared to fermented tiger nut beverage, which caused moderate depolarization but achieved superior cytotoxicity. We propose that mitochondrial dysfunction alone may not determine cytotoxic outcome; rather, the cellular capacity for antioxidant-mediated recovery may determine whether mitochondrial stress leads to adaptation or death. Unfermented tiger nut beverage, despite inducing severe acute mitochondrial stress, may allow cellular compensation through maintained GSH levels, potentially enabling recovery. Conversely, fermented tiger nut beverage may create sustained mitochondrial dysfunction in the context of compromised antioxidant defenses, potentially establishing a cellular environment where recovery becomes difficult and apoptotic pathways can progress to completion.

Among the compounds potentially responsible for these effects, ferulic acid, which was found at elevated levels in the fermented variant, has previously been reported to exert pro-oxidative effects by promoting ROS generation and inducing apoptosis in human lung cancer cells [23]. Therefore, the observed GSH depletion, in conjunction with enhanced ROS levels and mitochondrial dysfunction, supports the hypothesis that fermentation amplifies the pro-oxidant and pro-apoptotic potential of tiger nut beverage through the enrichment of specific bioactive metabolites. This suggests an oxidative stress hierarchy where fermented tiger nut beverage induces severe oxidative imbalance, unfermented tiger nut beverage produces compensated oxidative stress, and other treatments generate controlled oxidative perturbations that remain within cellular adaptive ranges.

However, it should be noted that fermented tiger nut showed significantly higher cytotoxicity toward normal CCD-18Co cells than toward cancer cells (*p* < 0.05), suggesting its evaluation as a general cytotoxic agent rather than a selective anticancer inducer, requiring targeted delivery systems or dose optimization to achieve tumor selectivity.

An important mechanistic insight emerges from the intracellular calcium analysis (Figure 7), which revealed that all tested beverages maintained normal cytosolic calcium levels despite inducing significant apoptosis, particularly in the case of fermented tiger nut beverage (Figure 2). This finding establishes that the observed antiproliferative effects operate through calcium-independent apoptotic pathways, consistent with previous studies using diindolylmethane, a bioactive compound derived from cruciferous vegetables, which demonstrated that alteration of Ca^2+^ homeostasis in the endoplasmic reticulum can induce cellular apoptosis through both calcium-dependent and calcium-independent mechanisms [25]. This pathway specificity could suggest a direct mitochondrial targeting mechanism rather than classical endoplasmic reticulum stress-mediated cell death. This has important therapeutic implications, as calcium-independent apoptotic mechanisms may exhibit enhanced selectivity by avoiding interference with calcium-dependent physiological processes in healthy tissues, potentially reducing off-target effects in clinical applications [25].

In contrast to the pronounced cytotoxic effects observed with the fermented tiger nut beverage, the carob beverages appeared to operate through a different mechanism. While its unfermented counterpart exhibited absence of effects on Caco-2 cell viability (Figure 1), the fermented carob beverage induced a clear arrest in the G_0_/G_1_ phase of the cell cycle (Figure 3), along with moderate mitochondrial membrane depolarization (Figure 6). These findings point toward a cytostatic rather than a cytotoxic response. This cytostatic effect is likely linked to the formation of specific flavonoids during fermentation, particularly luteolin and isorhamnetin, both of which have been previously identified in fermented carob beverage, but are not present in the unfermented form [4], (Supplementary Table S2). Luteolin has been shown to inhibit the proliferation of HT-29 colorectal cancer cells by inducing cell cycle arrest at the G_0_/G_1_ phase following short-term exposure (2 h), and at the G_2_/M phase after prolonged treatment (24 h), through modulation of cyclin-dependent kinases and checkpoint regulators [26]. Isorhamnetin, similarly, has demonstrated antiproliferative effects via cell cycle interference in G_2_/M transition, ROS overproduction and activation of mitochondrial apoptotic pathways [27].

Notably, our results showed that the fermented carob beverage did not induce significant ROS accumulation (Figure 4) or GSH depletion in Caco-2 cells, suggesting that oxidative stress was not a principal component of its moderate antiproliferative action. Despite the presence of mitochondrial membrane depolarization—often associated with early apoptotic signaling—the lack of oxidative markers points instead to a controlled, non-lethal modulation of mitochondrial function. This pattern supports the notion of a flavonoid-driven cytostatic mechanism, where the concentrations of luteolin and isorhamnetin present in the fermented beverage may have been sufficient to disrupt cell cycle progression without surpassing the pro-oxidant threshold required to initiate cell death.

Conversely, the rice-based beverages exhibited less consistent antiproliferative properties. Specifically, the unfermented rice beverage significantly reduced the viability of Caco-2 cells, while its fermented counterpart showed a noticeably diminished antiproliferative effect (Figure 1). This attenuation in antiproliferative activity following fermentation can likely be attributed to changes in the composition of phenolic compounds that occur during the fermentation process. In the fermented rice beverage analyzed in the present study, previously our group Vitali et al. [4], (Supplementary Table S3), reported a marked reduction in p-coumaric acid levels—an established phenolic compound known for its strong antiproliferative activity—alongside the emergence of new metabolites such as ethyl vanillin. Indeed, p-coumaric acid has been extensively studied for its ability to inhibit cancer cell proliferation in multiple colorectal cancer cell lines (i.e., DLD-1, HT-29, SW480, HTC-15, SW620 and Caco-2) through mechanisms such as induction of cell cycle arrest, apoptosis and modulation of oxidative stress [28]. Likewise, ethyl vanillin, a fermentation-derived compound, has also demonstrated antiproliferative effects in colorectal cancer models, although the available evidence is more limited [29]. Given the results observed in our study, the amount of ethyl vanillin generated during fermentation may not be sufficient to counterbalance the loss of p-coumaric acid. As a result, although the fermented rice beverage still contains bioactive components, the overall reduction in antiproliferative activity could likely be due to this quantitative and qualitative change in phenolic composition.

Taken together, these findings highlight the differential impact of fermentation on the antiproliferative properties of plant-based beverages, underscoring the importance of both the type of raw material and the resulting metabolite profile. It should be noted that the observed effects likely result from complex interactions among multiple bioactive compounds, including other phenolic compounds, limited number of peptides, and fermentation-derived metabolites, rather than individual compounds acting in isolation. While fermentation can enhance the bioactivity of certain matrices (e.g., tiger nut or carob beverages) by enriching them with potent pro-apoptotic and pro-oxidant compounds, though therapeutic benefit depends on achieving selective targeting of cancer cells (Table 1), it may also lead to the depletion of key phenolics, as observed in the case of rice, thereby reducing overall efficacy. The results establish a functional classification of these beverages: fermented tiger nut beverage as a potent cytotoxic agent operating through oxidative stress-mediated apoptosis, fermented carob beverage as a cytostatic agent utilizing flavonoid-driven cell cycle arrest, and rice beverages as moderate antiproliferative agents with matrix-dependent efficacy. These results emphasize the need for a targeted approach in the formulation of fermented functional beverages, taking into account the specific biochemical transformations induced by fermentation and digestion and their implications for CRC chemoprevention.

## 5. Conclusions

The present study highlights the differential antiproliferative responses of Caco-2 colorectal cancer cells to the BFs of fermented and unfermented plant-based beverages derived from rice, tiger nut and carob. Among these, the fermented tiger nut beverage exhibited the most potent cytotoxic effects, marked by enhanced apoptosis, mitochondrial membrane depolarization, ROS overproduction and GSH depletion, features strongly associated with the activation of the intrinsic apoptotic pathway. These effects were likely driven by fermentation-induced enrichment of bioactive compounds such as ferulic acid, known for its antiproliferative properties. In contrast, the fermented carob beverage primarily exerted cytostatic effects, inducing G0/G1 cell cycle arrest without triggering oxidative stress or significant apoptosis. This behavior is consistent with the presence of flavonoids such as luteolin and isorhamnetin, which modulate cell cycle progression through redox-independent mechanisms. Conversely, the fermented rice beverage showed a diminished antiproliferative effect relative to its unfermented counterpart, a phenomenon likely explained by the microbial degradation of p-coumaric acid and its replacement with less active metabolites. Taken together, these findings underscore the importance of fermentation and digestion as modulators of the bioactive profile and functional properties of plant-based beverages but also caution that its effects are highly matrix- and compound-specific.

Despite the valuable insights provided by this study, the use of a single in vitro model restricts the generalizability of the results, as it does not fully capture the complexity of tumor microenvironments. Additionally, the limited selectivity observed between tumor and normal cells, particularly with fermented tiger nut beverage, represents an important limitation that requires optimization of fermentation conditions to enhance therapeutic specificity. Future studies should therefore aim to validate these findings in more advanced biological models (e.g., tumor spheroids or organoids) and ultimately animal models of CRC. These models would allow for the assessment of bioavailability and potential synergistic interactions between fermented beverages and chemotherapeutic drugs. Such approaches will be essential to fully understand the therapeutic potential of fermented plant-based beverages as complementary strategies in CRC prevention and treatment.

## Figures and Tables

**Table 1:** Concentration of TSP and total antioxidant capacity present in the BFs of plant-based beverages used for antiproliferative effect in Caco-2 cells (diluted 1:15 *v*/*v* in culture medium), calculated from [4].

Bioaccessible Fraction						
Parameter	Tiger Nut		Carob		Rice	
	UF-D	F-D	UF-D	F-D	UF-D	F-D
Bioactive compounds						
(Total soluble polyphenols mg GAE/L)	0.190 ± 0.003 a	0.8 ± 0.1 b	0.0007 ± 0.0003 a	0.11 ± 0.04 b	1.6 ± 0.3 a	1.4 ± 0.3 a
Total antioxidant capacity						
ORAC (μM Trolox Eq/L)	94 ± 38 a	213 ± 19 b	419 ± 78 a	463 ± 111 a	176± 52 a	167 ± 60 a
TEAC (μM Trolox Eq/L)	219 ± 3 b	148± 11 a	115 ± 3 a	121 ± 4 a	124 ± 6 a	507 ± 27 b

Values are mean ± standard deviation (SD) (n = 3). Different letters (a–b) in the same row and for the same sample indicate significant differences (*p* < 0.05, ANOVA + Tukey test). UF-D: unfermented-digested; F-D: fermented-digested; GAE: gallic acid equivalents; ORAC: oxygen radical absorbance capacity; TEAC: Trolox equivalent antioxidant capacity.

## Data Availability

The original contributions presented in this study are included in the article/supplementary material. Further inquiries can be directed to the corresponding author.

## Supplementary Materials

The following supplementary information can be downloaded at: https://www.mdpi.com/article/10.3390/foods14173072/s1, Table S1: Peak integration (×10^6^) area values from the EICs of the phytochemical compounds identified by UHPLC-QTOF in non-fermented and fermented tiger nut beverages employing LAB consortia (VEGE061); Table S2: Peak integration (×10^6^) area values from the EICs of the phytochemical compounds identified by UHPLC-QTOF in non-fermented and fermented carob beverages employing LAB consortia (VEGE061); Table S3: Peak integration (×10^6^) area values from the EICs of the phytochemical compounds identified by UHPLC-QTOF in non-fermented and fermented rice beverages employing LAB consortia (VEGE061); Figure S1: Cytotoxicity assay of bioaccessible fractions from tiger nut beverages (T) and fermented tiger nut beverages (FT) beverages on Caco-2 cells after 24 h treatment; Figure S2: Cytotoxicity assay of bioaccessible fractions from carob beverages (C) and fermented carob beverages (FC) on Caco-2 cells after 24 h treatment; Figure S3. Cytotoxicity assay of bioaccessible fractions from rice beverages (R) and fermented rice beverages (FR) on Caco-2 cells after 24 h treatment. Cells were exposed to different dilutions (1:10, 1:12.5, 1:15, 1:17.5, 1:20 *v*/*v*). Cell viability was assessed by MTT assay and expressed as percentage relative to untreated control cells.
