# Supplementary material for: Differential Modulation of Cancer Cell Proliferation by Fermented Plant-Based Beverages: A Comparative Study of Tiger Nut, Carob and Rice Beverages in Colorectal Adenocarcinoma Cells
Source: Foods. 2025 Aug 30;14(17):3072. doi: 10.3390/foods14173072 (PMC12428345; doi:10.3390/foods14173072)
Supplement: Supplementary file 1 [file foods-14-03072-s001.zip › foods-3759350-supplementary.pdf]

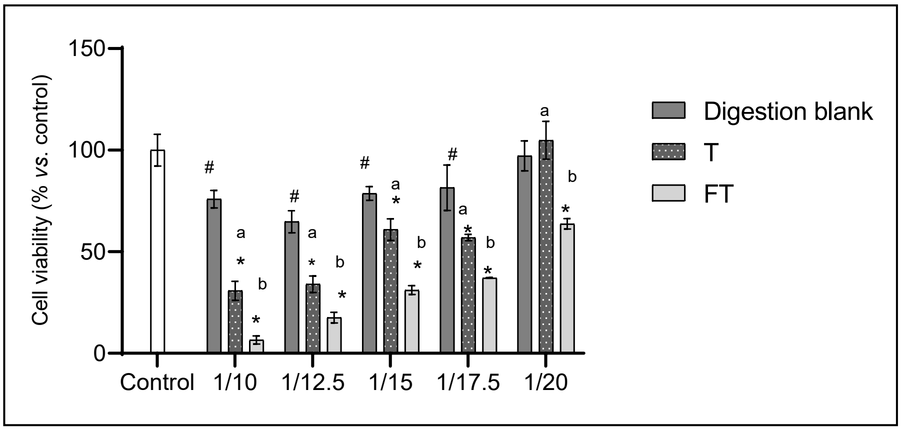

Figure S1. Cytotoxicity assay of bioaccessible fractions from tiger nut beverages (T) and fermented tiger nut beverages (FT) beverages on Caco-2 cells after 24 h treatment. Cells were exposed to different dilutions (1:10, 1:12.5, 1:15, 1:17.5, 1:20 v/v) Cell viability was assessed by MTT assay and expressed as percentage relative to untreated control cells. Data represent mean ± standard deviation (n=3). Statistical significance: # indicates significant differences compared to control; * indicates significant differences compared to digestion blank; different letters (a-b) indicate significant differences between non-fermented and fermented samples of the same matrix (Student's t-test; $p < 0.05$).

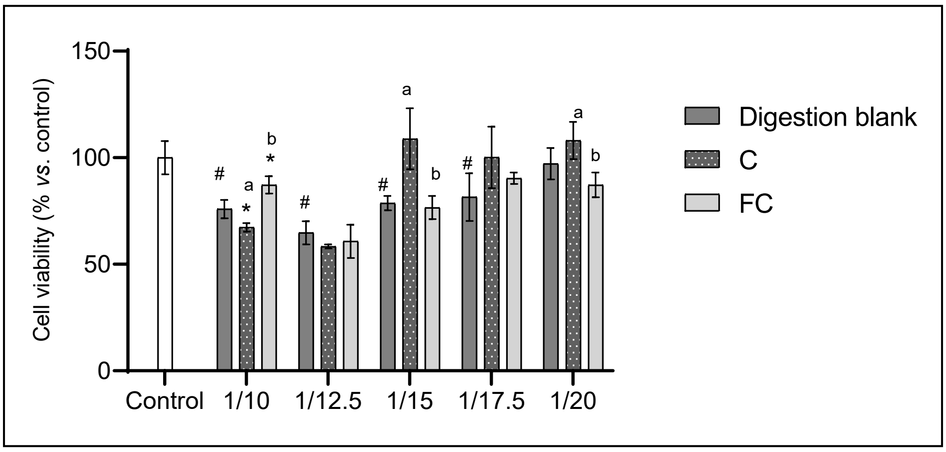

Figure S2. Cytotoxicity assay of bioaccessible fractions from carob beverages (C) and fermented carob beverages (FC) on Caco-2 cells after 24 h treatment. Cells were exposed to different dilutions (1:10, 1:12.5, 1:15, 1:17.5, 1:20 v/v). Cell viability was assessed by MTT assay and expressed as percentage relative to untreated control cells. Data represent mean ± standard deviation (n=3). Statistical significance: # indicates significant differences compared to control; * indicates significant differences compared to digestion blank; different letters (a-b) indicate significant differences between non-fermented and fermented samples of the same matrix (Student's t-test; $p < 0.05$).

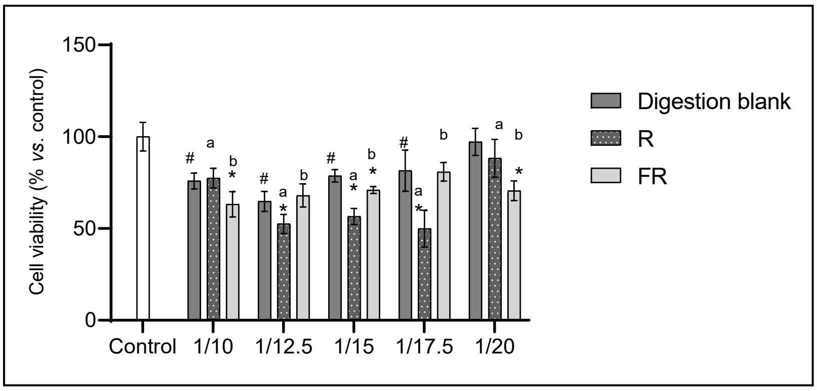

Figure S3. Cytotoxicity assay of bioaccessible fractions from rice beverages (R) and fermented rice beverages (FR) on Caco-2 cells after 24 h treatment. Cells were exposed to different dilutions (1:10, 1:12.5, 1:15, 1:17.5, 1:20 v/v). Cell viability was assessed by MTT assay and expressed as percentage relative to untreated control cells. Data represent mean ± standard deviation (n=3). Statistical significance: # indicates significant differences compared to control; * indicates significant differences compared to digestion blank; different letters (a-b) indicate significant differences between non-fermented and fermented samples of the same matrix (Student's t-test; $p < 0.05$).

Supplementary Material Discussion

Differences between samples and digestion blank and effect of dilution

All plant-based beverages demonstrated concentration-dependent cytotoxic effects on Caco-2 cells, with higher cytotoxicity observed at lower dilutions (1:10-1:12.5 v/v) and reduced effects at higher dilutions (1:17.5-1:20 v/v). Tiger nut beverages showed the most pronounced dilution-dependent response, maintaining significant cytotoxicity even at 1:15 v/v dilution (**figure S1**). Rice beverages exhibited moderate dilution sensitivity (**figure S3**), while carob beverages showed minimal cytotoxic effects across all tested dilutions compared to digestion blank controls (**figure S2**).

Differences due to fermentation

Fermentation effects varied markedly among plant matrices. Fermented tiger nut (FT) consistently enhanced cytotoxicity across all dilutions compared to non-fermented counterpart (T) (**figure S1**). Fermented carob (FC) showed no antiproliferative activity despite subtle improvements compared to non-fermented carob (C) (dilutions 1:15 and 1:20 v/v), though effects remained modest (**figure S2**). Conversely, fermented rice (FR) demonstrated reduced cytotoxic potential compared to non-fermented rice (R),

particularly evident from 1:12.5 v/v to 1:17.5 v/v dilutions, suggesting fermentation-induced degradation of bioactive compounds (**figure S3**).

Differences due to plant source

Plant matrix significantly influenced cytotoxic potential and dilution response patterns. Tiger nut beverages exhibited the strongest antiproliferative effects, maintaining significant activity even at higher dilutions (1:15-1:17.5 v/v) (**figure S1**). Rice beverages showed intermediate cytotoxicity with clear concentration dependence (**figure S3**). Carob beverages demonstrated the weakest cytotoxic effects across all dilutions, rarely achieving significant differences from digestion blank controls (**figure S2**). These matrix-specific responses reflect distinct bioactive compound profiles and their differential modulation during fermentation and digestion processes.

Table S1. Peak integration (x 10^6) area values from the EICs of the phytochemical compounds identified by UHPLC-QTOF in non-fermented and fermented tiger nut beverages employing LAB consortia (VEGE061).

Metabolites	Non-fermented	VEGE061
Homovanillic acid *	1.59±0.97 [a]	1.91±0.70 [a]
L-leucic acid	4.37±0.20 [a]	9.48±1.77 [b]
Ferulic acid *	n.d. [a]	3.62 ±0.43 [b]

* Phenolic compounds. EICs: Extracted Ion Chromatograms Metabolites. LAB: lactic acid bacteria. n.d.: not detected. Different letters (a-b) in the same row indicate statistically significant differences ($p < 0.05$) according to Welch's t-test.

Table S2. Peak integration (x 10^6) area values from the EICs of the phytochemical compounds identified by UHPLC-QTOF in non-fermented and fermented carob beverages employing LAB consortia (VEGE061).

Metabolites	Non-fermented	VEGE061
Luteolin *	n.d. [a]	96.96±0.70 [b]
**Isorhamnetin +***	n.d. [a]	19.87±3.26 [b] **(58.99±9.77 µM)**

* Phenolic compounds. EICs: Extracted Ion Chromatograms Metabolites. LAB: lactic acid bacteria. Metabolites highlighted in bold were quantified with external standard. n.d.: not detected. Different letters (a-b) in the same row indicate statistically significant differences ($p < 0.05$) according to Welch's t-test.

Table S3. Peak integration (x 10^6) area values from the EICs of the phytochemical compounds identified by UHPLC-QTOF in non-fermented and fermented rice beverages employing LAB consortia (VEGE061).

Metabolites	Non-fermented	VEGE061
p-coumaric acid *	1.91±0.12 [a]	0.86±0.13 [b]
Ethyl vanillin *	n.d. [a]	8.17±1.10 [b]

* Phenolic compounds. EICs: Extracted Ion Chromatograms Metabolites. LAB: lactic acid bacteria. n.d.: not detected. Different letters (a-b) in the same row indicate statistically significant differences ($p < 0.05$) according to Welch's t-test.

Note: For complete methodological details, full polyphenol profiles, and comprehensive analytical procedures used for compound identification and quantification, please refer to Vitali et al. (2025) [4].

Reference:

4. Vitali, M.; Gandía, M.; Garcia-Llatas, G.; González-Sarrías, A.; Vallejo, F.; Cilla, A.; Gamero, A. Modulation of antioxidant capacity, nutritional composition, probiotic viability after digestion and sensory attributes of plant-based beverages through lactic acid fermentation. *Foods* **2025**, *14*(9), 1447.
